# Electroacupuncture Combined with Cognitive Behavioral Therapy for Insomnia Alleviates Insomnia–Anxiety Comorbidity in Gynecological Cancer Patients Undergoing Chemotherapy: A Randomized Controlled Trial

**DOI:** 10.3390/curroncol33100628

**Published:** 2026-10-19

**Authors:** Ting Xu, Yanqiu Rao, Qi Cao, Yalin Yue

**Affiliations:** Department of Integrated Traditional Chinese and Western Medicine, Obstetrics and Gynecology Hospital of Fudan University, Shanghai 200090, China; xuting7685@fckyy.org.cn (T.X.); raoyanqiu6950@fckyy.org.cn (Y.R.)

**Keywords:** electroacupuncture, cognitive behavioral therapy for insomnia, gynecological cancer, chemotherapy, randomized controlled trial

## Abstract

Insomnia and anxiety affect many gynecological cancer patients on chemotherapy, and non-pharmacological options are limited. This randomized trial examined whether adding electroacupuncture (EA) to cognitive behavioral therapy for insomnia (CBT-I) improves both conditions. Seventy-six patients received EA + CBT-I or sham EA + CBT-I for 4 weeks. Outcomes included sleep quality (PSQI), insomnia severity (AIS), anxiety (HAMA), objective sleep parameters, and overall response rate. At Week 4, the EA group showed a greater reduction in PSQI global score (mean difference in change from baseline: −3.11, 95% CI: −4.11 to −2.11, *p* < 0.001). More patients in the EA group achieved AIS ≤ 6 (71.1% vs. 42.1%, *p* = 0.010) and HAMA ≤ 14 (92.1% vs. 65.8%, *p* = 0.005). Deep sleep (*p* = 0.001), REM sleep (*p* = 0.045), and sleep score (*p* = 0.008) also increased more with EA group. The overall response rate was higher with EA (92.1% vs. 71.1%, *p* = 0.016). No serious adverse events occurred. These findings suggest that adding EA to CBT-I improves sleep and anxiety in this population, with a favorable safety profile.

## Simple Summary

Insomnia and anxiety often occur together in gynecological cancer patients receiving chemotherapy. These symptoms reduce quality of life and can interfere with treatment. Cognitive behavioral therapy for insomnia (CBT-I) helps many patients, but responses vary. This randomized trial tested whether adding electroacupuncture (EA) to CBT-I could improve outcomes. Seventy-six patients received either EA plus CBT-I or sham EA plus CBT-I for 4 weeks. The real EA group had better sleep, less insomnia, and lower anxiety. More patients in this group achieved meaningful improvement. Sleep tracker data also indicated increases in deeper sleep and REM sleep. No serious adverse effects occurred. These findings suggest that adding EA to CBT-I is a safe and effective option for this population.

## 1. Introduction

Insomnia symptoms—difficulty falling asleep, staying asleep, or early morning awakening—affect 30% to 60% of cancer patients, well above the 10% to 20% observed in the general population [1,2]. These figures come mainly from self-reported symptom scales rather than structured diagnostic interviews, and they cover mixed cancer populations and treatment stages. Among gynecological cancer patients, the risk is even higher. Female sex, hormone changes after surgery, and chemotherapy neurotoxicity all play a role [3]. Poor sleep in these patients lowers quality of life, weakens immune function, and may reduce treatment adherence and worsen cancer outcomes [4]. Sleep and immunity also are also closely linked: poor sleep promotes inflammation, which may in turn drive tumor progression and treatment resistance [5].

Insomnia and anxiety frequently coexist in cancer patients, with comorbidity rates of 30–50%. Patients with both conditions generally have more severe symptoms and poorer treatment responses than those with either condition alone [6]. Some observational studies have also linked insomnia symptoms to poorer overall survival in cancer patients, although these findings are correlational and cannot establish causality [7]. Common mechanisms include HPA axis over-activity, GABA and serotonin dysregulation, and chronic low-grade inflammation [8]. This suggests that insomnia and anxiety reflect a shared underlying dysregulation and should be treated together rather than as separate problems [9].

Benzodiazepines and Z-drugs, commonly used for insomnia, carry risks of tolerance, dependence, daytime sedation, and interactions with chemotherapies [10]. These risks, together with many cancer patients’ preference for non-drug options, have driven interest in behavioral and mind–body approaches [11]. CBT-I is the first-line non-pharmacological treatment and has shown benefit in cancer survivors [12]. However, many patients do not achieve full remission with CBT-I alone. Its effect on co-occurring anxiety is limited, especially in the short term, and delivering CBT-I requires time and trained staff, which are often in short supply in oncology clinics [13].

Over the past 20 years, acupuncture has become increasingly integrated into cancer care, with evidence supporting its effects on pain, nausea, and fatigue, and newer data suggesting benefits for sleep and mood problems [14]. Electroacupuncture (EA) was selected over manual acupuncture for several reasons. EA provides standardized, reproducible stimulation parameters, which reduces variability between practitioners and enhances methodological rigor. A recent Bayesian network meta-analysis of acupuncture therapies for insomnia found that EA ranked favorably for improving sleep efficiency [15]. EA has also been investigated specifically for insomnia during systemic cancer therapy; a meta-analysis of randomized trials in breast cancer patients showed significant improvements in sleep outcomes [16]. Furthermore, EA has demonstrated anxiolytic effects in clinical and preclinical studies, potentially through modulation of HPA axis activity, GABAergic transmission, and neuroimmune pathways [17]. It has also been used safely during chemotherapy without evidence of increased adverse events or interference with chemotherapy efficacy.

Key questions remain. Few studies have focused specifically on acupuncture for insomnia–anxiety comorbidity in gynecological cancer patients receiving active chemotherapy [18,19]. Most existing trials have small samples, lack sham controls, or rely only on subjective measures [20]. It is also unclear whether adding acupuncture to CBT-I provides extra benefit—a question with direct clinical relevance.

To address these questions, we designed a randomized, sham-controlled trial based on the “Tiaoshen Ningxin” acupuncture principle. This traditional Chinese medicine framework focuses on regulating the “brain spirit” through Governor Vessel points (Baihui GV20, Shenting GV24) and calming the “heart spirit” through Heart Meridian points (Shenmen HT7), with the addition of Yintang (EX-HN3) and electroacupuncture applied at Baihui and Yintang. We hypothesized that this protocol, combined with CBT-I, would improve insomnia and anxiety more than sham acupuncture plus CBT-I. The primary aim was to assess efficacy and safety; secondary aims included objective sleep measurement with wearable trackers and exploration of possible mechanisms.

## 2. Materials and Methods

### 2.1. Study Design and Participants

This single-center randomized controlled trial was conducted at the Obstetrics and Gynecology Hospital of Fudan University (Shanghai, China) between May 2023 and August 2025. The trial was registered with the Chinese Clinical Trial Registry (ChiCTR2300071082) on 4 May 2023 and approved by the hospital’s Ethics Committee (2022-188) on 27 February 2023. All participants provided written informed consent. The trial was conducted in accordance with the Declaration of Helsinki and CONSORT reporting guidelines.

Eligible patients were 20 to 70 years of age with histologically confirmed gynecological malignancy (ovarian, cervical, endometrial, vulvar, vaginal, uterine sarcoma, or gestational trophoblastic neoplasia) and comorbid insomnia and anxiety. At enrollment, all patients were receiving active chemotherapy. Randomization and baseline assessments took place during a scheduled chemotherapy cycle, and the 4-week intervention period overlapped with ongoing chemotherapy. Insomnia was diagnosed using the Chinese Guidelines for the Diagnosis and Treatment of Insomnia in Adults (2017) [21]. Anxiety was defined as a HAMA score > 14, indicating clinically significant anxiety symptoms [22]. A formal DSM-5 diagnosis of generalized anxiety disorder was not required. Key exclusion criteria were use of sleep medications within 2 weeks before enrollment, severe organ dysfunction or psychiatric disorders unrelated to the primary cancer diagnosis, and concurrent participation in another interventional trial.

### 2.2. Randomization and Blinding

Eligible participants were randomly assigned in a 1:1 ratio to either the EA + CBT-I group or the sham EA + CBT-I group. The randomization sequence was computer-generated (SPSS 21.0) by an independent biostatistician and concealed in sequentially numbered, opaque, sealed envelopes. The acupuncturists who delivered the treatment could not be masked to group assignment. However, outcome assessors—who administered the PSQI, AIS, and HAMA scales, collected sleep tracker data, and documented adverse events—remained blinded to allocation, as did the statistician who performed the final analysis.

### 2.3. Intervention Protocol

All participants received standard chemotherapy and routine supportive care according to institutional protocols.

CBT-I (both groups): Both groups received a 4-week CBT-I program delivered in weekly sessions by trained therapists. The CBT-I program followed the American Academy of Sleep Medicine (AASM) definition, comprising sleep hygiene education, stimulus control, sleep restriction therapy, cognitive therapy, and relaxation therapy. Sleep restriction was adjusted based on daily sleep diaries. A recent systematic review and meta-analysis of 19 randomized controlled trials (*n* = 1803) supports the efficacy of CBT-I in cancer survivors [23].

EA group: Patients received electroacupuncture three times per week (every other day) for 4 weeks (12 sessions total). Four acupoints were selected based on the “Tiaoshen Ningxin” (regulating the spirit and calming the heart) principle: Baihui (GV20) and Shenting (GV24) on the Governor Vessel, which connects to the brain; Yintang (EX-HN3) to calm the spirit; and Shenmen (HT7), the Yuan-source point of the Heart Meridian, traditionally used for insomnia and anxiety. Acupoint locations followed the GB/T 12346-2021 standard [24]. Sterile disposable needles (0.25 mm × 25 mm; Suzhou Medical Supplies Factory Co., Ltd., Suzhou, China) were used. Shenmen was needled perpendicularly to a depth of 7–10 mm; the remaining points were inserted at a 15° angle to a depth of 10–20 mm. Manual stimulation was applied to elicit deqi. Electroacupuncture (SDZ-V, Hwato^®^, Suzhou, China) was then applied to Baihui and Yintang using a continuous wave at 2 Hz, with intensity adjusted to each patient’s comfort (typically 1–4 mA), for 30 min per session. A similar acupoint combination has been reported to reduce HPA axis activity in patients with chronic insomnia, lowering serum CRH, ACTH, and CORT levels [25]. Electroacupuncture at Baihui and Yintang also appears to modulate activity in sleep-related brain regions [17,26].

Sham EA group: The control group received sham electroacupuncture on the same schedule and at the same points. We placed blunt-tip placebo needles, identical in appearance to real needles, over the skin through adhesive pads without penetrating it. The needles were connected to an electroacupuncture device via internally disconnected leads; the device appeared active but delivered no current. Patients were told they might not feel the stimulation because it was a “microcurrent”.

Co-interventions: Additional sleep medications (including benzodiazepines, Z-drugs, and sedating antihistamines), acupuncture, and psychological therapy were not allowed during the study. We permitted analgesics (including opioids), antidepressants, and anxiolytics if they had been started at least 4 weeks before enrollment and the dose remained stable throughout the study period. We recorded any changes in these medications. Chemotherapy and supportive care medications (e.g., antiemetics, corticosteroids) were allowed as clinically indicated and were recorded.

### 2.4. Outcome Measures

All outcome assessments were carried out at baseline, Week 2, and Week 4 by assessors blinded to group allocation. The primary outcome was change in PSQI global score from baseline to Week 4. The PSQI is a 19-item self-report questionnaire with seven component scores (0–3 each) and a global score (0–21); higher scores indicate poorer sleep quality [27]. Because sleep medication use was an exclusion criterion at baseline and was not permitted during the study, we excluded the sleep medication component from the global score. The modified global score therefore ranged from 0 to 18. This modification is consistent with prior research that used a modified PSQI in populations with restricted hypnotic use [28]. Item response theory analyses have shown that the sleep medication component has the lowest discrimination parameter of the seven PSQI components, which supports excluding it when medication use is not relevant [29]. This avoids inflating the global score due to medication use and ensures that the score reflects sleep quality and disturbance rather than pharmacological treatment. The Cronbach’s α for the modified PSQI (6 components) was 0.72 in this study.

Secondary outcomes included: Insomnia severity was measured using the Athens Insomnia Scale (AIS), an 8-item self-report scale with a total score ranging from 0 to 24 [30]. Scores above 6 indicate insomnia, 4–6 suspected insomnia, and below 4 no insomnia [30,31]. Internal consistency for the AIS was not calculated in this study; however, prior validation studies have reported a Cronbach’s α of approximately 0.90 [30]. Anxiety was assessed with the Hamilton Anxiety Rating Scale (HAMA), a 14-item clinician-rated instrument with a total score ranging from 0 to 56 [32]. Scores above 14 indicate clinical anxiety, 7–14 suspected anxiety, and below 7 no anxiety [33]. Internal consistency for the HAMA was not calculated in this study; however, prior validation studies have reported a Cronbach’s α of approximately 0.89 [34]. The HAMA has also been validated in cancer populations [35]. Objective sleep parameters were recorded using a HUAWEI Band 8 (Huawei Device Co., Ltd., Shenzhen, China) worn on the non-dominant wrist throughout the study. The device uses the HUAWEI TruSleep 3.0 algorithm to estimate sleep stages and generate a sleep score (0–100), with higher scores indicating better sleep quality. This specific device has not been validated against polysomnography in a clinical population; however, the HUAWEI TruSleep algorithm has been evaluated in other Huawei wearable models, showing acceptable agreement with polysomnography for sleep/wake detection in Chinese clinical populations [36]. Measurements were obtained at baseline, Week 2, and Week 4. Comprehensive efficacy was graded according to the PSQI reduction rate, calculated as [(baseline total score—post-treatment total score)/baseline total score] × 100%. We classified efficacy as clinically cured (reduction ≥ 75%, nocturnal sleep ≥ 6 h, no disturbing dreams, and refreshed awakening); markedly effective (50% ≤ reduction < 75%, sleep improved with ≥3 h increase); effective (25% ≤ reduction < 50%, symptoms alleviated with <3 h increase); or ineffective (reduction < 25%, no improvement or worsening). The overall response rate was the proportion of patients who were clinically cured, markedly effective, or effective. Safety outcomes included adverse events (needle breakage, bending, fainting, hematoma, and infection), which were recorded at each session. Patients rated treatment acceptability on a 5-point scale after each session.

### 2.5. Sample Size Calculation

Sample size was determined for a superiority design with a 1:1 allocation ratio. We used a two-sided test with α = 0.05 and power (1 − β) = 0.90, corresponding to Z_1_ − α/_2_ = 1.96 and Z_1_ − β = 1.28. The formula was: n_1_ = n_2_ = 2 × [(Z_1_ − α/_2_ + Z_1_ − β)^2^ × σ^2^]/δ^2^. Based on previous studies, we assumed a minimum clinically important difference of 2.5 points in PSQI global score between groups (δ = 2.5) and a standard deviation of 3.0 (σ = 3.0), which yielded 35 participants per group. Accounting for a 10% dropout rate, we planned to enroll 38 participants per group, for a total of 76 participants.

### 2.6. Statistical Analysis

All analyses were performed with SPSS 21.0 (IBM Corp., Armonk, NY, USA). Statistical significance was set at a two-sided *p* < 0.05. We assessed normality using the Shapiro–Wilk test. Normally distributed continuous data are presented as mean ± SD and compared using independent-samples *t*-tests. Non-normally distributed data are presented as median with interquartile range and were analyzed using the Mann–Whitney U test. Categorical variables were compared using the chi-square test or Fisher’s exact test, as appropriate.

Between-group differences were calculated using change from baseline scores for the primary outcome (change in PSQI global score from baseline to Week 4) and its component scores. For secondary outcomes (AIS, HAMA, sleep tracker parameters), between-group differences were calculated using Week 4 scores. Within-group changes are reported separately as mean change from baseline.

### 2.7. Ethical Approval and Consent

The study was carried out in accordance with the Declaration of Helsinki. The protocol was approved by the Ethics Committee of the Obstetrics and Gynecology Hospital of Fudan University (Approval No. 2022-188) on 27 February 2023. Written informed consent was obtained from every participant before enrollment.

### 2.8. Data Availability

The datasets generated and analyzed during this study are not publicly available due to privacy and ethical restrictions. Deidentified data supporting the findings reported in this manuscript may be requested from the corresponding author, subject to institutional review board approval and execution of a formal data use agreement. This study was reported in line with the CONSORT 2025 guidelines [37]; the completed checklist is provided as Supplementary Material.

### 2.9. Use of Generative AI

During the preparation of this manuscript, the authors used DeepSeek (DeepSeek-V3-0324, 2025) for language editing, formatting, proofreading, and reference realignment. The authors have reviewed and edited the output and take full responsibility for the content of this publication.

## 3. Results

### 3.1. Participant Flow and Baseline Characteristics

**Figure 1:**
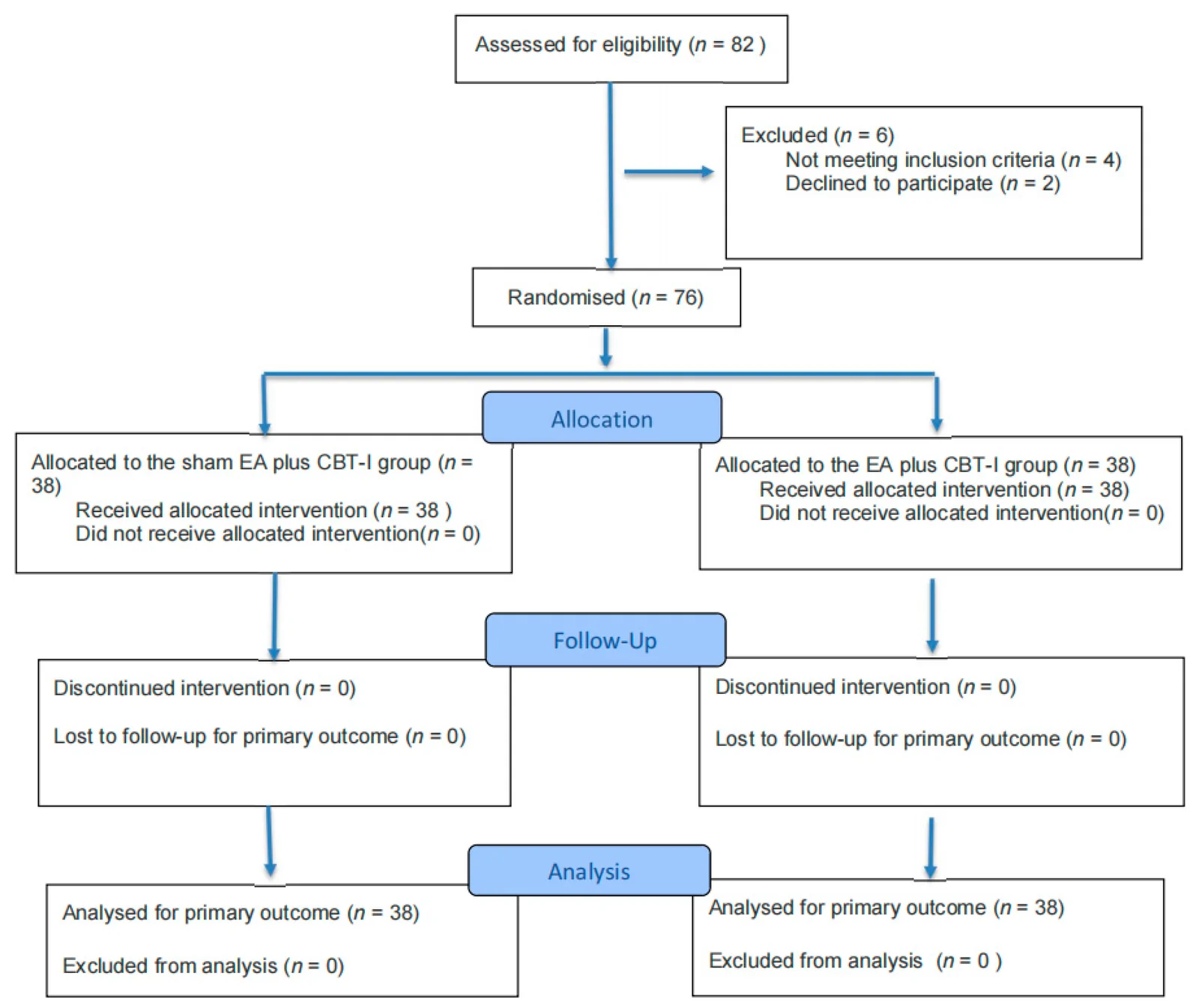
CONSORT flow diagram of the study. A total of 82 patients were assessed for eligibility; 6 were excluded (4 did not meet inclusion criteria, 2 declined to participate). A total of 76 patients were randomized: 38 to the EA + CBT-I group and 38 to the sham EA + CBT-I group. All 76 patients completed the 4-week intervention and follow-up and were included in the intention-to-treat analysis.

**Figure 2:**
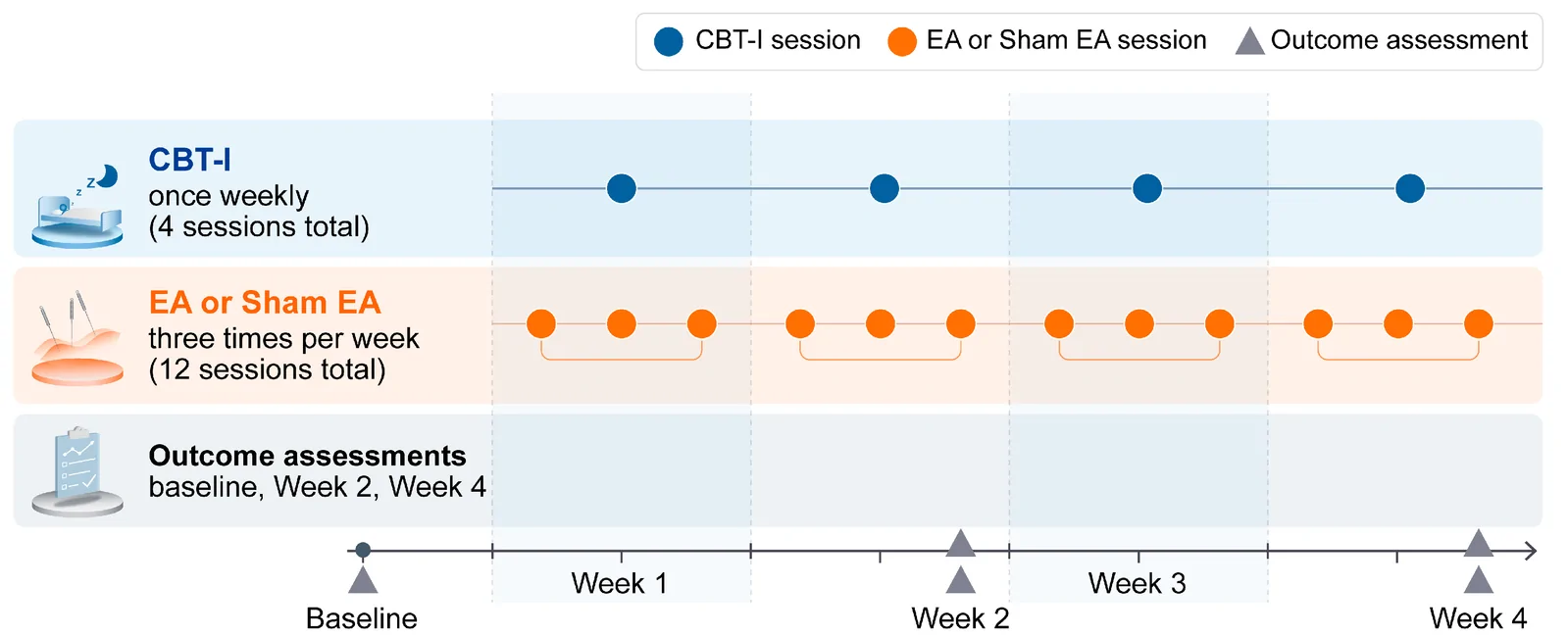
Standard treatment schedule for a single patient over the 4-week study period. CBT-I sessions were given once weekly (Weeks 1–4). Electroacupuncture (EA) or sham EA was given three times per week (every other day) for 4 consecutive weeks (12 sessions total). Outcome assessments were performed at baseline, Week 2, and Week 4.

Between May 2023 and August 2025, we screened 82 patients for eligibility. Six were excluded: four did not meet the inclusion criteria and two declined to participate. The remaining 76 patients were randomized, 38 to the EA + CBT-I group and 38 to the sham EA + CBT-I group. All 76 completed the 4-week intervention and follow-up, with no dropouts (Figure 1). The standard treatment schedule is shown in Figure 2. Baseline characteristics did not differ between the two groups (all *p* > 0.05; Table 1). Cancer types were balanced between groups: ovarian/fallopian tube (*n* = 24 vs. 21), cervical (*n* = 9 vs. 12), endometrial (*n* = 4 vs. 3), and other gynecological malignancies (*n* = 1 vs. 2) in the control and experimental groups, respectively (χ^2^ = 2.11, *p* = 0.550). Tumor stage distribution was also similar between groups (Stage I–II: 8 vs. 6; Stage III: 18 vs. 17; Stage IV/recurrent: 12 vs. 15; χ^2^ = 0.65, *p* = 0.723), as was radiotherapy history (14 vs. 16; χ^2^ = 0.22, *p* = 0.640). All patients were receiving active chemotherapy at enrollment.

### 3.2. Primary Outcome

At Week 4, the mean change from baseline in PSQI global score was −8.24 ± 2.05 in the EA group and −5.13 ± 2.32 in the control group, yielding a between-group difference of −3.11 (95% CI: −4.11 to −2.11, *p* < 0.001). The EA group also had a lower PSQI global score than the control group (6.97 ± 2.56 vs. 9.84 ± 3.12). All six PSQI components showed greater improvement with the EA group (all *p* < 0.05), with the largest differences in sleep latency (−0.63, 95% CI: −0.89 to −0.37; *p* < 0.001) and sleep efficiency (−0.58, 95% CI: −0.85 to −0.31; *p* < 0.001) (Table 2).

### 3.3. Secondary Outcomes

At Week 4, the EA group had lower AIS scores than the control group (5.39 ± 2.87 vs. 8.63 ± 3.56; between-group difference based on Week 4 scores: −3.24, 95% CI: −4.72 to −1.76; *p* < 0.001). More patients in the EA group achieved AIS ≤ 6 (71.1% vs. 42.1%, *p* = 0.010) and AIS < 4 (28.9% vs. 10.5%, *p* = 0.044) (Table 3). HAMA scores were also lower in the EA group at Week 4 (7.68 ± 3.85 vs. 13.55 ± 5.34; between-group difference based on Week 4 scores: −5.87, 95% CI: −8.06 to −3.68; *p* < 0.001), with more patients reaching HAMA ≤ 14 (92.1% vs. 65.8%, *p* = 0.005) and HAMA < 7 (50.0% vs. 13.2%, *p* < 0.001) (Table 4).

Sleep tracker data were consistent with the subjective findings. The EA group had larger increases in deep sleep (34.2 ± 14.6 vs. 15.4 ± 12.8 min; between-group difference 18.7 min, 95% CI: 8.0 to 29.4; *p* = 0.001), REM sleep (21.0 ± 14.2 vs. 11.2 ± 13.5 min; between-group difference 10.5 min, 95% CI: 0.3 to 20.7; *p* = 0.045), and overall sleep score (6.3 ± 4.8 vs. 2.6 ± 4.2; between-group difference 3.4 points, 95% CI: 0.9 to 5.9; *p* = 0.008). Light sleep increased in both groups, with no between-group difference (P = 0.288) (Table 5).

The mean PSQI reduction rate was higher in the EA group than in the control group (54.6% ± 16.8% vs. 33.8% ± 14.2%), with a between-group a difference of 20.8 percentage points (95% CI: 13.7 to 27.9; *p* < 0.001). The efficacy grade distribution was more favorable in the EA group (Mann–Whitney U test: Z = −3.72, *p* < 0.001). The overall response rate was 92.1% (35/38) in the EA group versus 71.1% (27/38) in the control group (*p* = 0.016), and the marked response rate (clinically cured + markedly effective) was 65.8% (25/38) versus 31.6% (12/38) (*p* = 0.003) (Table 6).

### 3.4. Safety and Tolerability

No serious adverse events—such as needle breakage, bending, fainting, or infection—occurred in either group. Mild local bruising at the acupoint site occurred in one patient in each group and resolved without intervention. Treatment acceptability was high in both groups. Concomitant medication use was recorded for all participants. No patient started a new sleep medication or changed the dose of existing psychotropic medications during the study. We did not compare concomitant medication use between groups quantitatively, as this was not a prespecified outcome. Because doses remained stable and no new sleep medications were started, these medications likely had limited influence on the primary outcomes.

## 4. Discussion

Adding EA to CBT-I for 76 gynecological cancer patients on chemotherapy with insomnia and anxiety improved sleep quality, insomnia severity, and anxiety compared with sham acupuncture plus CBT-I. Benefits were consistent across subjective scales, objective sleep parameters, and comprehensive efficacy ratings. The intervention was safe and well tolerated.

The between-group difference in PSQI change from baseline to Week 4 (−3.11, 95% CI: −4.11 to −2.11) exceeded the MCID of 2.5 points assumed in our sample size calculation [38]. However, the MCID for the PSQI has not been universally established, and this threshold should be viewed as a reference rather than a definitive criterion. The EA group‘s mean PSQI score decreased to 6.97, while the control group remained above 9.84. Because we used a modified PSQI excluding the sleep medication component, the conventional cutoff of 5 should be interpreted with caution. We therefore focus on the between-group difference and the within-group change rather than the proportion of patients below a specific cutoff. The NNT of 5 for overall response and 3 for marked response suggest that the intervention is clinically efficient. These findings are in line with meta-analyses showing that acupuncture improves sleep in cancer patients [16,39], and they extend the evidence by demonstrating an incremental benefit when EA is added to CBT-I, rather than comparing the two as alternatives. This likely reflects complementary mechanisms: EA addresses neurobiological and physiological aspects, while CBT-I targets maladaptive sleep cognitions and behaviors [40].

Insomnia and anxiety improved in parallel in the EA group, suggesting that EA may act on shared pathways underlying both conditions, which supports the principle of treating the comorbidity as a whole [9]. CBT-I alone has only modest effects on anxiety [41], whereas our data indicate that adding EA boosts this effect. This is relevant for cancer patients, in whom insomnia and anxiety share common triggers: diagnosis-related distress, treatment side effects, and fear of recurrence. They also share neurobiological underpinnings such as HPA axis overactivity and GABAergic dysfunction [8]. Targeting both at once may be more efficient than treating each symptom separately.

Sleep tracker data suggested greater increases in deep sleep and REM sleep in the EA group. However, the HUAWEI Band 8 has not been independently validated for sleep staging in cancer patients, and wearable devices generally show lower accuracy in patients with insomnia than in healthy sleepers. These sleep-stage findings should therefore be considered exploratory and hypothesis-generating rather than confirmatory. Still, the direction of change was consistent with the subjective reports, which reduces concern about measurement bias [42,43]. Our data do not establish the mechanisms underlying these effects. We did not measure HPA axis activity, GABAergic or serotonergic transmission, or inflammatory markers in this trial. The acupoints used in this study have been linked to modulation of these pathways in previous preclinical and clinical studies. A randomized trial using the same acupoints (Baihui, Shenting, Yintang, Shenmen) in chronic insomnia reported significant reductions in serum CRH, ACTH, and CORT, suggesting downregulation of HPA axis hyperactivity [25]. Shenmen (HT7) has been associated with increased GABAergic neurotransmission and modulation of serotonergic activity in preclinical studies. Electroacupuncture has also been shown to normalize HPA axis activity, enhance GABAergic transmission, suppress neuroimmune activation [17], and reduce peripheral pro-inflammatory cytokines. However, whether these mechanisms apply to gynecological cancer patients undergoing chemotherapy remains unclear. Future studies with biomarker assessments are needed to test these hypotheses.

No serious adverse events occurred in either group. The low incidence of minor side effects—only occasional mild bruising—combined with no drug interactions and good acceptability, makes this approach attractive for cancer patients, who often take multiple medications and prefer non-drug options [11]. This safety profile compares well with pharmacological alternatives, which carry risks of tolerance, dependence, and daytime sedation [10,44].

The acupoints used in this study (Baihui, Shenting, Yintang, and Shenmen) were chosen for their established effects on sleep and emotional regulation, not for any direct anti-tumor effect. Traditionally, these points are used to calm the mind and promote sleep. In our trial, their clinical benefits were seen in insomnia and anxiety, which were the primary therapeutic targets. Whether acupuncture at these points may also affect cancer-related outcomes through modulation of the neuro-endocrine–immune network remains unclear and needs further study.

Because both groups received CBT-I, we cannot determine whether EA adds benefit beyond CBT-I alone. The comparison was between EA plus CBT-I and sham EA plus CBT-I, so the specific effect of EA and any non-specific effects of the acupuncture procedure remain indistinguishable. A three-arm trial with a CBT-I-only group would be needed to resolve this question.

This study has several limitations. First, it was a single-center trial with a relatively short follow-up, so the findings should be considered preliminary. It is also unclear whether the effects last beyond 4 weeks or require booster sessions. Second, the acupuncturists could not be blinded, although participants and outcome assessors were masked to allocation. Third, we did not include a CBT-I-only arm, so we cannot fully isolate the specific contribution of EA beyond CBT-I. Fourth, the HUAWEI Band 8 has not been independently validated for sleep staging in cancer patients, and wearable devices generally show lower accuracy in patients with insomnia. Fifth, anxiety was defined by HAMA score > 14 rather than by a structured diagnostic interview for DSM-5 generalized anxiety disorder; the findings should therefore be interpreted in the context of anxiety symptom severity. Sixth, the study population included multiple gynecological cancer types with differing disease biology, stage, and treatment characteristics. Cancer type, tumor stage, and radiotherapy history were balanced between groups, but detailed information on chemotherapy regimens, cumulative cycles, and corticosteroid exposure was not systematically recorded, and concomitant medication use was not quantitatively analyzed. The sample size (*n* = 76) also limited our ability to perform extensive multivariable adjustment. These factors can independently affect sleep and anxiety and may have influenced the outcomes. Seventh, we used a modified PSQI excluding the sleep medication component; although this approach is justified by the study design, the PSQI was originally validated as a full instrument. Finally, we did not collect long-term oncologic or survival data, so the impact of improved sleep and anxiety on cancer outcomes remains unknown. Larger, multi-center trials with longer follow-up and comprehensive treatment data are needed to confirm these findings.

Overall, adding electroacupuncture to CBT-I improved insomnia, anxiety, and objective sleep in this population, with consistent benefits across subjective and objective measures and good tolerability. These findings support further investigation of acupuncture-based interventions in supportive cancer care.

## 5. Conclusions

Adding electroacupuncture to CBT-I may improve insomnia, anxiety, and objective sleep quality in gynecological cancer patients undergoing chemotherapy, with a good safety profile. These benefits may involve shared neurobiological pathways underlying insomnia and anxiety, such as HPA axis activity, GABAergic and serotonergic transmission, and inflammatory signaling. However, given the short follow-up and single-center design, these findings are preliminary. Larger, multi-center trials with biomarker assessments are needed to confirm long-term efficacy and clarify mechanisms.

## Figures and Tables

**Table 1:** Baseline demographic and clinical characteristics of participants.

	Control Group (*n* = 38)	Experimental Group (*n* = 38)	Test Statistic	*p*
Demographics				
Age (years), mean ± SD	54.3 ± 9.8	53.8 ± 10.2	t = 0.22	0.828
Height (cm), mean ± SD	159.2 ± 5.9	158.5 ± 6.5	t = −0.49	0.626
Weight (kg), mean ± SD	59.1 ± 11.1	58.7 ± 10.3	t = −0.16	0.872
Disease duration (months), M (P25, P75)	24.0 (4.0, 78.0)	36.0 (5.0, 60.0)	Z = −0.23	0.820
Education level, *n* (%)			χ^2^ = 0.22	0.974
Primary school or below	8 (21.1%)	8 (21.1%)		
Junior high school	14 (36.8%)	12 (31.6%)		
High school	9 (23.7%)	10 (26.3%)		
College degree or above	7 (18.4%)	8 (21.1%)		
Radiotherapy history, *n* (%)			χ^2^ = 0.22	0.640
Yes	14 (36.8%)	16 (42.1%)		
No	24 (63.2%)	22 (57.9%)		
Cancer type, *n* (%)			χ^2^	*p*
Ovarian/fallopian tube	24 (63.2%)	21 (55.3%)	2.11	0.55
Cervical	9 (23.7%)	12 (31.6%)		
Endometrial	4 (10.5%)	3 (7.9%)		
Other	1 (2.6%)	2 (5.3%)		
Tumor stage, *n* (%)			χ^2^ = 0.65	0.723
Stage I–II	8 (21.1%)	6 (15.8%)		
Stage III	18 (47.4%)	17 (44.7%)		
Stage IV/Recurrent	12 (31.6%)	15 (39.5%)		
Baseline scale scores				
AIS score, mean ± SD	13.74 ± 2.95	14.11 ± 2.87	t = 0.55	0.585
HAMA score, mean ± SD	23.42 ± 6.89	24.08 ± 7.24	t = 0.41	0.683

AIS = Athens Insomnia Scale; HAMA = Hamilton Anxiety Rating Scale; SD = standard deviation. Continuous variables are presented as mean ± standard deviation (SD) for normally distributed data or as median (P25, P75) for nonnormally distributed data. Normally distributed variables (age, height, weight, AIS, HAMA) were compared using independent-samples *t*-tests; the non-normally distributed variable (disease duration) was compared using Mann–Whitney U test. Categorical variables are presented as counts (percentages) and were compared using Pearson’s chi-square test. All tests were two-sided, and *p* < 0.05 was considered statistically significant.

**Table 2:** PSQI component scores and global scores at each time point.

Component	Group	Baseline	Week 2	Week 4	Within-Group Change	Between-Group Difference in Change from Baseline (95% CI)	*p* *
Global PSQI score							
	Control	14.97 ± 2.71	12.13 ± 2.94	9.84 ± 3.12	−5.13 ± 2.32 ^a^		
	Experimental	15.21 ± 2.45	10.82 ± 2.89	6.97 ± 2.56	−8.24 ± 2.05 ^a^	−3.11 (−4.11, −2.11)	<0.001
Subjective sleep quality							
	Control	2.26 ± 0.64	1.76 ± 0.63	1.42 ± 0.68	−0.84 ± 0.55 ^a^		
	Experimental	2.32 ± 0.62	1.53 ± 0.60	0.97 ± 0.59	−1.35 ± 0.54 ^a^	−0.51 (−0.76, −0.26)	<0.001
Sleep latency							
	Control	2.42 ± 0.59	2.03 ± 0.59	1.63 ± 0.63	−0.79 ± 0.58 ^a^		
	Experimental	2.47 ± 0.55	1.76 ± 0.59	1.05 ± 0.61	−1.42 ± 0.55 ^a^	−0.63 (−0.89, −0.37)	<0.001
Sleep duration							
	Control	2.29 ± 0.70	1.89 ± 0.76	1.55 ± 0.72	−0.74 ± 0.64 ^a^		
	Experimental	2.34 ± 0.63	1.71 ± 0.73	1.08 ± 0.63	−1.26 ± 0.64 ^a^	−0. 52 (−0.81, −0.23)	0.001
Sleep efficiency							
	Control	2.39 ± 0.59	2.05 ± 0.61	1.63 ± 0.67	−0.76 ± 0.59 ^a^		
	Experimental	2.45 ± 0.55	1.82 ± 0.61	1.11 ± 0.69	−1.34 ± 0.58 ^a^	−0.58 (−0.85, −0.31)	<0.001
Sleep disturbance							
	Control	1.68 ± 0.57	1.45 ± 0.55	1.18 ± 0.56	−0.50 ± 0.56 ^a^		
	Experimental	1.76 ± 0.54	1.34 ± 0.53	0.89 ± 0.51	−0.87 ± 0.53 ^a^	−0.37 (−0.62, −0.12)	0.004
Daytime dysfunction							
	Control	2.92 ± 0.27	2.68 ± 0.47	2.42 ± 0.68	−0.50 ± 0.51 ^a^		
	Experimental	2.87 ± 0.34	2.29 ± 0.61	1.87 ± 0.70	−1.00 ± 0.53 ^a^	−0.50 (−0.74, −0.26)	0.001

CI = confidence interval; PSQI = Pittsburgh Sleep Quality Index. Values are presented as mean ± SD. Within-group changes are presented as mean change ± SD. Between-group differences in all PSQI metrics (global score and individual components) were calculated based on change from baseline scores (Week 4—Baseline). ^a^ *p* < 0.001 vs. baseline within the same group (paired *t* test). * *p* value for between-group comparison at Week 4 using independent-samples *t*-test. All normally distributed variables (Shapiro–Wilk test, *p* > 0.05) with homogeneous variances (Levene’s test, *p* > 0.05). All tests were two-sided; *p* < 0.05 was considered statistically significant.

**Table 3:** Athens insomnia scale (AIS) outcomes.

Outcome	Group	Baseline	Week 2	Week 4	Change (Week 2-Baseline)	Change (Week 4-Baseline)	*p* *
AIS total score							
	Control	13.74 ± 2.95	11.08 ± 3.45	8.63 ± 3.56	−2.66 ± 2.02 ^a^	−5.11 ± 2.45 ^a^	
	Experimental	14.11 ± 2.87	9.45 ± 3.21	5.39 ± 2.87	−4.66 ± 1.79 ^a^	−8.71 ± 1.94 ^a^	
	Between-group difference (95% CI)	0.37 (−0.94, 1.68)	−1.63 (−3.15, −0.11)	−3.24 (−4.72, −1.76)	−2.00 (−3.07, −0.93) ^b^	−3.60 (−4.63, −2.57) ^b^	<0.001
AIS categorical outcomes at Week 4							
		Control (*n* = 38)	Experimental (*n* = 38)				
	AIS ≤ 6 (no/suspected insomnia), *n* (%)	16 (42.1%)	27 (71.1%)				0.010 ^c^
	AIS < 4 (no insomnia), *n* (%)	4 (10.5%)	11 (28.9%)				0.044 ^c^

AIS = Athens Insomnia Scale; CI = confidence interval; SD = standard deviation. Continuous data are presented as mean ± SD. Within-group changes are presented as mean change ± SD. Categorical data are presented as *n* (%). AIS interpretation: Total score ranges from 0–24; <4 = no insomnia, 4–6 = suspected insomnia, >6 = insomnia. ^a^ *p* < 0.001 vs. baseline within the same group (paired *t* test). * *p* for between-group comparison at Week 4 (independent-samples *t*-test for continuous variables; Pearson’s chi-square test for categorical variables). ^b^ *p* < 0.001 for between-group comparison of change magnitudes (independent-samples *t*-test). ^c^ *p* for between-group comparison of proportions (Pearson’s chi-square test). All continuous variables followed a normal distribution (Shapiro–Wilk, *p* > 0.05) with homogeneous variances (Levene’s, *p* > 0.05). All tests were two-sided; *p* < 0.05 was considered statistically significant.

**Table 4:** Hamilton anxiety rating scale (HAMA) outcomes.

Outcome	Group	Baseline	Week 2	Week 4	Change (Week 2—Baseline)	Change (Week 4—Baseline)	*p* *
HAMA total score							
	Control	23.42 ± 6.89	18.42 ± 5.97	13.55 ± 5.34	−5.00 ± 3.08 ^a^	−9.87 ± 3.41 ^a^	
	Experimental	24.08 ± 7.24	14.76 ± 4.63	7.68 ± 3.85	−9.32 ± 3.98 ^a^	−16.39 ± 4.51 ^a^	
	Between-group difference (95% CI)	0.66 (−2.52, 3.84)	−3.66 (−6.09, −1.23)	−5.87 (−8.06, −3.68)	−4.32 (−5.96, −2.68) ^b^	−6.52 (−8.36, −4.68) ^b^	<0.001
HAMA categorical outcomes							
		Time point	Control (*n* = 38)	Experimental (*n* = 38)			
	HAMA ≤ 14 (no/suspected anxiety), *n* (%)	Week 2	6 (15.8%)	21 (55.3%)			<0.001 ^c^
		Week 4	25 (65.8%)	35 (92.1%)			0.005 ^c^
	HAMA < 7 (no anxiety), *n* (%)	Week 2	0 (0%)	1 (2.6%)			0.314 ^d^
		Week 4	5 (13.2%)	19 (50.0%)			<0.001 ^c^

CI = confidence interval; HAMA = Hamilton Anxiety Rating Scale; SD = standard deviation. Continuous data are presented as mean ± SD. Within-group changes are presented as mean change ± SD. Categorical data are presented as *n* (%). HAMA interpretation: Total score ranges from 0–56; <7 = no anxiety, 7–14 = suspected anxiety, >14 = clinical anxiety. ^a^ *p* < 0.001 vs. baseline within the same group (paired *t*-test). * *p* for between-group comparison at Week 4 (independent-samples *t*-test for continuous variables; Pearson’s chi-square test for categorical variables). ^b^ *p* < 0.001 for between-group comparison of change magnitudes (independent-samples *t*-test). ^c^ *p* for between-group comparison of proportions (Pearson’s chi-square test). ^d^ Fisher’s exact test (expected cell count < 5). All continuous variables followed a normal distribution (Shapiro–Wilk, *p* > 0.05) with homogeneous variances (Levene’s, *p* > 0.05). All tests were two-sided; *p* < 0.05 was considered statistically significant.

**Table 5:** Sleep monitoring tracker outcomes at each time point.

Outcome	Group	Baseline	Week 2	Week 4	Within-Group Change (Week 4-Baseline)	Between-Group Difference at Week 4 (95% CI)	*p* *
Deep sleep (min)							
	Control	104.1 ± 18.4	112.8 ± 19.7	119.5 ± 22.4	15.4 ± 12.8 ^a^		
	Experimental	104.0 ± 19.8	121.9 ± 22.1	138.2 ± 25.3	34.2 ± 14.6 ^a^	18.7 (8.0, 29.4)	0.001
Light sleep (min)							
	Control	205.3 ± 44.8	227.4 ± 42.3	235.7 ± 43.8	30.4 ± 28.7 ^a^		
	Experimental	207.1 ± 46.7	241.8 ± 40.5	246.3 ± 42.5	39.2 ± 31.2 ^a^	10.6 (−8.9, 30.1)	0.288
REM sleep (min)							
	Control	102.7 ± 20.1	110.2 ± 22.6	113.9 ± 22.3	11.2 ± 13.5 ^a^		
	Experimental	103.4 ± 21.4	115.5 ± 21.3	124.4 ± 22.8	21.0 ± 14.2 ^a^	10.5 (0.3, 20.7)	0.045
Sleep score							
	Control	76.8 ± 7.2	78.5 ± 6.7	79.4 ± 6.1	2.6 ± 4.2 ^a^		
	Experimental	76.5 ± 6.8	80.2 ± 5.9	82.8 ± 4.8	6.3 ± 4.8 ^a^	3.4 (0.9, 5.9)	0.008

CI = confidence interval; min, minutes; REM = rapid eye movement; SD = standard deviation. Values are presented as mean ± SD. Within-group changes are presented as mean change ± SD. Deep sleep reflects sleep recovery function; REM sleep reflects memory consolidation function; Sleep score ranges from 0–100, with higher scores indicating better overall sleep quality. ^a^ *p* < 0.001 vs. baseline within the same group (paired *t*-test). * *p* for between-group comparison at Week 4 (independent-samples *t*-test). All continuous variables followed a normal distribution (Shapiro–Wilk, *p* > 0.05) with homogeneous variances (Levene’s, *p* > 0.05). All tests were two-sided; *p* < 0.05 was considered statistically significant.

**Table 6:** Comprehensive efficacy outcomes based on PSQI reduction rate.

Efficacy Outcome	Control Group (*n* = 38)	Experimental Group (*n* = 38)	Test Statistic	*p*
PSQI reduction rate (%), mean ± SD	33.8 ± 14.2	54.6 ± 16.8	t = 5.82	<0.001
Efficacy grade distribution, *n* (%)			Z = −3.72	<0.001 ^b^
Clinically cured (≥75%)	2 (5.3%)	5 (13.2%)		
Markedly effective (50% to <75%)	10 (26.3%)	18 (47.4%)		
Effective (25% to <50%)	15 (39.5%)	12 (31.6%)		
Ineffective (<25%)	11 (28.9%)	3 (7.9%)		
Overall response rate (cured + markedly effective + effective)	27 (71.1%)	35 (92.1%)	χ^2^ = 5.78	0.016
Marked response rate (cured + markedly effective)	12 (31.6%)	25 (65.8%)	χ^2^ = 8.94	0.003

PSQI = Pittsburgh Sleep Quality Index; SD = standard deviation. Continuous data are presented as mean ± SD. Categorical data are presented as *n* (%). Efficacy was graded based on the PSQI reduction rate: Reduction rate = [(Baseline total score—Posttreatment total score)/Baseline total score] × 100%. ^b^ Mann–Whitney U test for ordered categorical efficacy grade distribution. PSQI reduction rate (continuous variable) was compared using independent samples *t* test (normal distribution confirmed by Shapiro–Wilk test, *p* > 0.05; homogeneous variances confirmed by Levene’s test, *p* > 0.05). Overall response rate and marked response rate (binary variables) were compared using Pearson’s chi-square test. All tests were two-sided; *p* < 0.05 was considered statistically significant.

## Data Availability

The datasets generated and/or analyzed during the current study are available from the corresponding author upon reasonable request.

## Supplementary Materials

The following supporting information can be downloaded at: https://www.mdpi.com/article/10.3390/curroncol33100628/s1, The CONSORT 2025 Statement is cited in the main text as reference [37].

## Abbreviations

The following abbreviations are used in this manuscript:

AIS	Athens Insomnia Scale
CBT-I	Cognitive behavioral therapy for insomnia
CI	Confidence interval
CONSORT	Consolidated Standards of Reporting Trials
EA	Electroacupuncture
HAMA	Hamilton Anxiety Rating Scale
IQR	Interquartile range
ITT	Intention to treat
PSQI	Pittsburgh Sleep Quality Index
REM	Rapid eye movement
SD	Standard deviation

## References

[1] Savard J., Morin C.M. (2001). Insomnia in the context of cancer: A review of a neglected problem. J. Clin. Oncol..

[2] Otte J.L., Carpenter J.S., Manchanda S., Rand K.L., Skaar T.C., Weaver M., Chernyak Y., Zhong X., Igega C., Landis C. (2015). Systematic review of sleep disorders in cancer patients: Can the prevalence of sleep disorders be ascertained?. Cancer Med..

[3] Pozzar R.A., Hammer M.J., Paul S.M., Cooper B.A., Kober K.M., Conley Y.P., Levine J.D., Miaskowski C. (2021). Distinct sleep disturbance profiles among patients with gynecologic cancer receiving chemotherapy. Gynecol. Oncol..

[4] Palesh O., AldridgeGerry A., Zeitzer J.M., Koopman C., Neri E., GieseDavis J., Jo B., Kraemer H., Nouriani B., Spiegel D. (2014). Actigraphymeasured sleep disruption as a predictor of survival among women with advanced breast cancer. Sleep.

[5] Besedovsky L., Lange T., Born J. (2012). Sleep and immune function. Pflugers Arch..

[6] Taylor D.J., Lichstein K.L., Durrence H.H., Reidel B.W., Bush A.J. (2005). Epidemiology of insomnia, depression, and anxiety. Sleep.

[7] Savard J., Villa J., Ivers H., Simard S., Morin C.M. (2009). Prevalence, natural course, and risk factors of insomnia comorbid with cancer over a 2 month period. J. Clin. Oncol..

[8] Riemann D., Krone L.B., Wulff K., Nissen C. (2020). Sleep, insomnia, and depression. Neuropsychopharmacology.

[9] Nutt D., Wilson S., Paterson L. (2008). Sleep disorders as core symptoms of depression. Dialogues Clin. Neurosci..

[10] Morin C.M., Benca R. (2012). Chronic insomnia. Lancet.

[11] Garland S.N., Johnson J.A., Savard J., Gehrman P., Perlis M., Carlson L., Campbell T. (2014). Sleeping well with cancer: A systematic review of cognitive behavioral therapy for insomnia in cancer patients. Neuropsychiatr. Dis. Treat..

[12] Garland S.N., Carlson L.E., Stephens A.J., Antle M.C., Samuels C., Campbell T.S. (2014). Mindfulnessbased stress reduction compared with cognitive behavioral therapy for the treatment of insomnia comorbid with cancer: A randomized, partially blinded, noninferiority trial. J. Clin. Oncol..

[13] Morin C.M., Vallières A., Guay B., Ivers H., Savard J., Mérette C., Bastien C., Baillargeon L. (2009). Cognitive behavioral therapy, singly and combined with medication, for persistent insomnia: A randomized controlled trial. JAMA.

[14] Zhang X.W., Hou W.B., Pu F.L., Wang X.F., Wang Y.R., Yang M., Cheng K., Wang Y., Robinson N., Liu J.P. (2022). Acupuncture for cancer related conditions: An overview of systematic reviews. Phytomedicine.

[15] Liu L., Chen Y., Tian M., Wu Z., Zhang P., Zhang T., Yang J., Qu H., Fan Z., Wu S. (2025). The efficacy and safety of multiple acupuncture therapies in primary insomnia: A Bayesian network meta-analysis. Integr. Med. Res..

[16] Gao L., Sun Y., Luo T., Chen H., Huang S., Zhu L., Ye M. (2025). Acupuncture for systemic therapy-associated insomnia in patients with breast cancer: A systematic review and meta-analysis of randomized controlled trials. Front. Oncol..

[17] Wang X., Yan L., Zhang X., Liu X., Yang B. (2025). Electroacupuncture as adjunctive therapy for insomnia via targeting the GABAergic microbiota-gut-brain axis: A mini review. Front. Psychiatry.

[18] Kwag E., Li X., Garland S., Bryl K., Taylor L., Li Q.S., Amann L., Mao J.J., McConnell K.M. (2025). Acupuncture versus cognitive behavioral therapy for anxiety among cancer survivors with insomnia: An exploratory analysis of a randomized clinical trial. Integr. Med. Res..

[19] Garland S.N., Xie S.X., DuHamel K., Bao T., Li Q., Barg F.K., Song S., Kantoff P., Gehrman P., Mao J.J. (2019). Acupuncture Versus Cognitive Behavioral Therapy for Insomnia in Cancer Survivors: A Randomized Clinical Trial. J. Natl. Cancer Inst..

[20] Cheuk D.K., Yeung W.F., Chung K.F., Wong V. (2012). Acupuncture for insomnia. Cochrane Database Syst. Rev..

[21] Chinese Society of Neurology, Sleep Disorders Group of Chinese Society of Neurology (2018). Chinese guidelines for the diagnosis and treatment of insomnia in adults (2017). Chin. J. Neurol..

[22] American Psychiatric Association (2013). Diagnostic and Statistical Manual of Mental Disorders.

[23] Cooper J.T., Svoboda E., Prochazka A.V., Ha D.M. (2025). Effects of cognitive-behavioral therapy for insomnia compared with controls among cancer survivors: A systematic review and meta-analysis of randomized trials. BMC Cancer.

[24] (2021). Nomenclature and Location of Meridian Points.

[25] Xi H.Q., Wu W.Z., Liu C.Y., Wang X.Q., Zhao Y.N., Zheng S.Y., Li J.H. (2020). Effect of Tongdu Tiaoshen needling in treatment of chronic insomnia by regulating the hypothalamic-pituitary-adrenal axis. Zhen Ci Yan Jiu.

[26] Zhang G., Qu S., Zheng Y., Chen J., Deng G., Yang C., Huang Y. (2013). Key regions of the cerebral network are altered after electroacupuncture at the Baihui (GV20) and Yintang acupuncture points in healthy volunteers: An analysis based on resting fcMRI. Acupunct. Med..

[27] Buysse D.J., Reynolds C.F., Monk T.H., Berman S.R., Kupfer D.J. (1989). The Pittsburgh Sleep Quality Index: A new instrument for psychiatric practice and research. Psychiatry Res..

[28] Hinz A., Friedrich M., Schulte T., Petrowski K., Tibubos A.N., Hartung T.J. (2025). The Pittsburgh Sleep Quality Index (PSQI) Applied to Cancer Patients: Psychometric Properties and Factors Affecting Sleep Quality. Cancer Investig..

[29] Zhu B., Xie M., Park C.G., Kapella M.C. (2018). Adaptation of the Pittsburgh Sleep Quality Index in Chinese adults with type 2 diabetes. J. Chin. Med. Assoc..

[30] Soldatos C.R., Dikeos D.G., Paparrigopoulos T.J. (2000). Athens Insomnia Scale: Validation of an instrument based on ICD-10 criteria. J. Psychosom. Res..

[31] Soldatos C.R., Dikeos D.G., Paparrigopoulos T.J. (2003). The diagnostic validity of the Athens Insomnia Scale. J. Psychosom. Res..

[32] Hamilton M. (1959). The assessment of anxiety states by rating. Br. J. Med. Psychol..

[33] Matza L.S., Morlock R., Sexton C., Malley K., Feltner D. (2010). Identifying HAM-A cutoffs for mild, moderate, and severe generalized anxiety disorder. Int. J. Methods Psychiatr. Res..

[34] Lobo A., Chamorro L., Luque A., Dal-Ré R., Badia X., Baró E., Grupo de Validación en Español de Escalas Psicométricas (GVEEP) (2002). Validación de las versiones en español de la Montgomery-Asberg Depression Rating Scale y la Hamilton Anxiety Rating Scale para la evaluación de la depresión y de la ansiedad [Validation of the Spanish versions of the Montgomery-Asberg depression and Hamilton anxiety rating scales]. Med. Clin..

[35] Hendi M., Zhang B., Lv J.M., Cai X.J. (2025). Factors influencing anxiety and depression in advanced hepatocellular carcinoma patients and their impact on quality of life. World J. Psychiatry.

[36] Guo J., Luo J., Xiao Y., Huang R. (2025). Validity and clinical utility of a wrist-worn device against polysomnography. PLoS ONE.

[37] Hopewell S., Chan A.W., Collins G.S., Hróbjartsson A., Moher D., Schulz K.F., Tunn R., Aggarwal R., Berkwits M., Berlin J.A. (2025). CONSORT 2025 statement: Updated guideline for reporting randomised trials. BMJ.

[38] Qin Z., Zhu Y., Shi D.D., Chen R., Li S., Wu J. (2024). The gap between statistical and clinical significance: Time to pay attention to clinical relevance in patient-reported outcome measures of insomnia. BMC Med. Res. Methodol..

[39] Khosravaninezhad Y., Moshfeghinia R., Liaghat L., Ghazipoor H., Kaheni Y., Javidan A., Mirzaee Y., Azadmehr A., Hashemi Y., Pasalar M. (2025). Effect of acupressure and acupuncture on sleep quality in cancer patients with insomnia: A systematic review and metaanalysis. Explore.

[40] Xing J., Wu X., Liu H., Wang J., Jiang S., Lozada A., Wang Y. (2020). Effects of Electroacupuncture Therapy and Cognitive Behavioral Therapy in Chronic Insomnia: A Randomized Controlled Study. Evid. Based Complement. Alternat Med..

[41] Belleville G., Cousineau H., Levrier K., St-Pierre-Delorme M.È. (2011). Metaanalytic review of the impact of cognitivebehavior therapy for insomnia on concomitant anxiety. Clin. Psychol. Rev..

[42] Besedovsky L., Lange T., Haack M. (2019). The SleepImmune Crosstalk in Health and Disease. Physiol. Rev..

[43] Harvey A.G., Tang N.K. (2012). (Mis) perception of sleep in insomnia: A puzzle and a resolution. Psychol. Bull..

[44] Krystal A., Benca R., McCall W.V., Perlis M.L., Watson N.F., Winkelman J.W., Fisher M., Morin C.M. (2026). Pharmacologic treatment data from chronic insomnia studies and the application to individuals with acute insomnia, a narrative review. Sleep Med. Rev..

